# Slug inhibits the proliferation and tumor formation of human cervical cancer cells by up-regulating the p21/p27 proteins and down-regulating the activity of the Wnt/β-catenin signaling pathway via the trans-suppression Akt1/p-Akt1 expression

**DOI:** 10.18632/oncotarget.8434

**Published:** 2016-03-28

**Authors:** Nan Cui, Wen-Ting Yang, Peng-Sheng Zheng

**Affiliations:** ^1^ Department of Reproductive Medicine, First Affiliated Hospital, Xi'an Jiaotong University Medical School, Xi'an, The People's Republic of China; ^2^ Department of Biochemistry and Molecular Biology, Xi'an Jiaotong University Medical School, Xi'an, The People's Republic of China; ^3^ Section of Cancer Stem Cell Research, Key Laboratory of Environment and Genes Related to Diseases, Ministry of Education of The People's Republic of China, Xi'an, The People's Republic of China

**Keywords:** Slug, Akt1, Wnt/β-catenin, cervical cancer, proliferation

## Abstract

Slug (Snai2) has been demonstrated to act as an oncogene or tumor suppressor in different human cancers, but the function of Slug in cervical cancer remains poorly understood. In this study, we demonstrated that Slug could suppress the proliferation of cervical cancer cells *in vitro* and tumor formation *in vivo*. Further experiments found that Slug could trans-suppress the expression of Akt1/p-Akt1 by binding to E-box motifs in the promoter of the Akt1 gene and then inhibit the cell proliferation and tumor formation of cervical cancer cells by up-regulating p21/p27 and/or down-regulating the activity of the Wnt/β-catenin signaling pathway. Therefore, Slug acts as a tumor suppressor during cervical carcinogenesis.

## INTRODUCTION

Globally, cervical carcinoma is the third most common tumor type and the fourth most common cause of cancer death among women [1]. In developing countries, cervical carcinoma is the second most common gynecological cancer and the second most common cause of cancer death among women [2]. Nearly 99.7% of cervical cancer cases are associated with the human papillomavirus (HPV) [3–5]. However, the molecular and genetic mechanisms involved in the initiation and progression of cervical cancer remain poorly understood. Previous studies observed that various oncogenes and cancer suppressor genes exhibit abnormal expression during the development and progression of cervical cancer. For example, the oncogene R-Ras is reported to play a central role during the progression of cervical cancer [6]. p53 is known to function as a tumor suppressor in various tumors, but p53 polymorphisms were reported to be associated with an increased risk of cervical cancer [7, 8]. Recently, accumulating evidence showed that some stem cell self-renewal-associated transcription factors are involved in tumorigenesis and tumor development in cervical cancer; for example, NANOG, OCT4, Msi1 and LGR5 are reported to promote the progression of cervical cancer [9–12]; in contrast, UTF1 and KLF4 are reported to function as tumor suppressors in cervical cancer [13, 14]. In addition, SOX2 was reported to enhance tumor formation ability and to serve as a nuclear marker for cervical cancer stem cells [15], and ALDH1 was reported to be a cytoplasmic maker for cervical cancer stem cells [16].

Slug is a member of the Snail superfamily, and evidence indicates that Slug is associated with cell pluripotency [17, 18]. When Slug and Sox9 are coexpressed in mammary stem cells, these proteins help to maintain the mammary stem cell population [19]. In addition, during mammary gland morphogenesis, Slug controls the growth dynamics of stem/progenitor cells as a gate-keeper [20]. Early studies suggested that Slug is implicated in the development of chick limbs and involved in the early patterning of the mesoderm and the neural crest [21]. On the other hand, the expression of Slug is an initial and necessary step for epithelial to mesenchymal transition (EMT) and plays a critical role during the EMT program [22–24]. As a transcriptional repressor, Slug regulates the expression of target genes by binding to E-box elements in the promoter regions of genes. For instance, Slug can induce the reduction of E-cadherin in various cancers [25–27], and this transcription repression capacity was stabilized by p19Arf in mouse prostate cancer models [28]. In addition, the down-regulation of the cell adhesion molecule E-cadherin is also one of the most important changes for EMT [29], and E-cadherin is also associated with the self-renewal of human embryonic stem cells (hESC) [30, 31]. Moreover, as an antiapoptotic factor, Slug is also beneficial for cell survival [32–34].

However, the function of Slug in cervical cancers is poorly understood. Early studies showed that Slug exerts contrasting effects on cell proliferation and tumor growth among various cancers; for example, Slug promotes cell proliferation in lung cancer cells and glioblastoma cells [35, 36] but inhibits cell proliferation in human epidermal keratinocytes and human prostate cancer cells [37, 38]. This study reported for the first time that Slug could inhibit proliferation and tumor formation in human cervical cancer cells by up-regulating p21/p27 and/or down-regulating cyclin D1 via the trans-suppression of Akt1/p-Akt1.

## RESULTS

### The expression of slug in the normal human cervix and different cancerous cervical lesions

**Figure 1:**
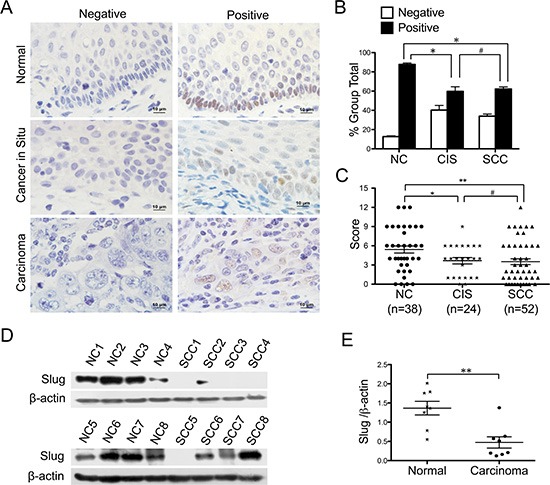
Expression of slug in normal cervix samples and various cervical lesions (**A**) Immunohistochemical (IHC) detection of Slug in normal cervix, cancer *in situ* and carcinoma samples; original magnification, 1000×. (**B**) Slug staining is classified into 2 categories (negative and positive), and the bar chart shows the percentage of each group (38 normal cervix specimens, 24 carcinoma *in situ* specimens, and 52 invasion carcinoma tissue specimens). (**C**) The scatter plot shows the immunoreactivity scores (IHC) obtained for the staining of Slug in normal cervix, cervical cancer *in situ* and invasive cervical cancer samples (points represent the IHC score per specimen, and one-way ANOVA was performed). (**D**) The expression of Slug in normal cervix (NC) and squamous cervical carcinoma (SCC) samples was detected using western blotting. (**E**) The relative expression of Slug in each normal cervix tissue (*n* = 8) and squamous cervical carcinoma tissue sample (*n* = 8) is shown. The data shown are the ratios of the Slug/β-actin of each specimen and the means ± standard error of the NC and SCC groups (triangles represent relative Slug expression). Values are shown as the mean ± SD, *p < 0.05, **p < 0.01.

To investigate whether Slug is involved in the development and progression of human cervical carcinoma, Slug expression was detected in normal human cervix (NC), cervical cancer *in situ* (CIS) and invasive cervical cancer (SCC) samples using immunohistochemistry. Representative Slug staining in the normal cervix and cancerous cervical lesions are shown in Figure 1A. The Slug protein was found to localize to the nucleus. The Slug-positive rate decreased from NC samples (86.84%) to CIS samples (62.50%) and SCC samples (59.62%). Significant differences were observed between CIS and NC samples and between SCC and NC samples, but no significant difference was observed between CIS and SCC samples (Table S1 and Figure 1B, *p* < 0.05). The immunoreactivity scores were also lower in CIS and SCC samples than in NC samples (Figure 1C, CIS vs. NC, *P* < 0.05; SCC vs. NC, *p* < 0.01), but there was no significant difference between the CIS and SCC samples (Figure 1C), suggesting that Slug is involved in the development of cervical carcinoma. Additionally, western blotting was used quantitatively to detect the expression of Slug in 8 normal cervix samples and 8 cervical carcinoma samples (Figure 1D). The average Slug expression level was lower in cervical carcinoma tissues than in normal cervix tissues (Figure 1E; *P* < 0.01), further confirming that Slug expression is negatively related to cervical carcinogenesis.

### Slug inhibits the proliferation of cervical carcinoma cells *in vitro*

**Figure 2:**
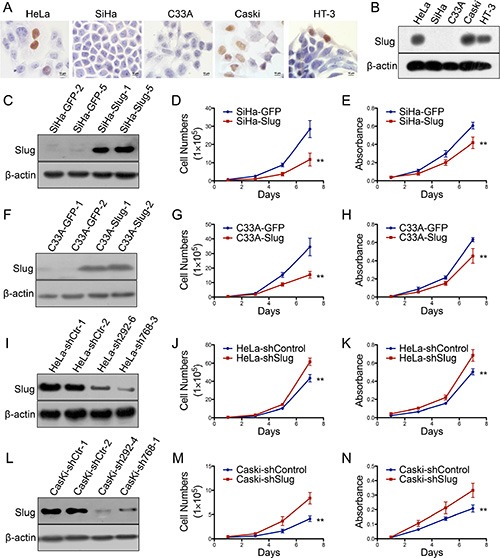
Slug inhibits the proliferation of human cervical cancer cell lines *in vitro* Slug expression in human cervical cancer cell lines was detected using immunocychemistry (**A**) and western blotting (**B**). Stably transfected cell lines were identified by western blotting: (**C**) SiHa-GFP and SiHa-Slug cells; (**F**) C33A-GFP and C33A-Slug cells; (**I**) HeLa-shControl and HeLa-shSlug cells; and (**L**) Caski-shControl and Caski-shSlug cells. The proliferation and viability of SiHa-GFP and SiHa-Slug cells were detected using growth curves (**D**) and the MTT assay (**E**). The proliferation and viability of C33A-GFP and C33A-Slug cells were detected using growth curves (**G**) and the MTT assay (**H**). The proliferation and viability of HeLa-shControl and HeLa-shSlug were detected using growth curves (**J**) and the MTT assay (**K**). The proliferation and viability of Caski-shControl and Caski-shSlug cells were detected using growth curves (**M**) and the MTT assay (**N**). The data were shown as the mean ± SD of three independent experiments. *p < 0.05, **p < 0.01 vs. control using One-Way ANOVA.

Moreover, the expression of Slug was detected using immunocytochemistry and western blotting in cervical cancer cell lines. A high level of Slug expression was detected in the HeLa, CasKi and HT-3 cervical carcinoma cell lines, but almost no expression of the Slug protein was detected in SiHa and C33A cells (Figure 2A and 2B). To further investigate the function of Slug in human cervical cancer cells, exogenous Slug was stably overexpressed in SiHa (SiHa-Slug, Figure 2C) and C33A (C33A-Slug, Figure 2F) cells; conversely, the expression of Slug was knocked down in HeLa Figure HeLa-shSlug, Figure 2I) and CasKi (CasKi-shSlug, Figure 2L) cells by stably transfecting shRNA plasmids. Western blotting was used to confirm the effects of the up-regulation and down-regulation of Slug expression in all cervical cancer cells (SiHa, C33A, HeLa and CasKi) and their controls (Figure 2C, 2F, 2I and 2L, respectively).

Cell growth curves and the MTT assay were used to determine the cell proliferation ability and cell viability of the Slug-modified cervical cancer cell lines and their control cells. As shown in Figure 2D and 2G, the SiHa-Slug and C33A-Slug cells grew much more slowly than their respective control cells (SiHa-GFP and C33A-GFP, *p* < 0.01). In addition, the viability of SiHa-Slug and C33A-Slug cells was also much lower than that of their respective control cells (SiHa-GFP and C33A-GFP) (Figure 2E and 2H; *p* < 0.01), suggesting that the Slug protein may suppress the proliferation of cervical cancer cells. Furthermore, both cell growth curves and cell viability assays found that HeLa-shSlug and CasKi-shSlug cells grow much faster than their respective control cells (HeLa-shcontrol and Caski-shcontrol) (Figure 2J, 2M, Figure 2K and 2N; *p* < 0.01), suggesting that the knockdown of Slug promoted the proliferation of cervical cancer cells. All of these results demonstrated that the Slug protein inhibited the proliferation of cervical carcinoma cells *in vitro*.

### Slug suppresses the growth and tumor formation of cervical cancer cells *in vivo*

**Figure 3:**
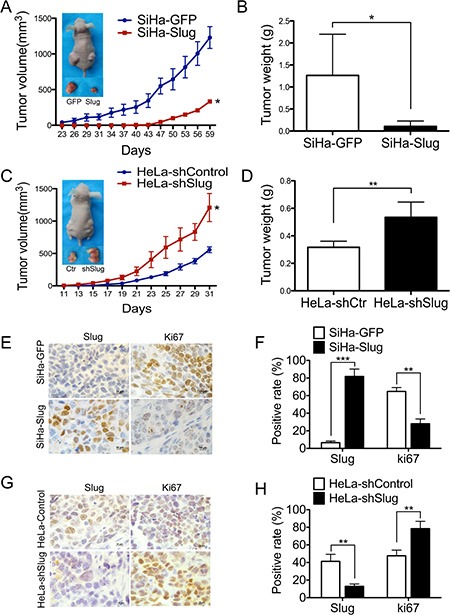
Slug suppressed cervical carcinoma tumor growth *in vivo* Tumor growth curves were calculated after injection into female nude mice based on monitoring performed every 3 days: (**A**) SiHa-GFP and SiHa-Slug cells; (**C**) HeLa-shControl and HeLa-shSlug cells. The xenograft tumors were dissociated and weighed at the end of experiment: (**B**) SiHa-GFP and SiHa-Slug cells; (**D**) HeLa-shControl and HeLa-shSlug cells. Immunohistochemical staining of Slug and Ki-67 in xenograft tumor tissues: (**E** and **F**) SiHa-GFP and SiHa-Slug cells; (**G** and **H**) HeLa-shControl and HeLa-shSlug cells. Values are shown as the mean ± SD. *p < 0.05, **p < 0.01, ***p < 0.001 *vs*. control using One-Way ANOVA.

To identify the effect of Slug on cervical cancer cells *in vivo*, 10^6^ Slug-modified cells and their control cells were inoculated subcutaneously into female nude mice for the tumor formation assay. Both SiHa and HeLa cells formed xenografted tumors successfully. However, the C33A and CasKi cells failed to form palpable tumors in female nude mice. As shown in Figure 3A, the palpable tumors formed by SiHa-GFP control cells could be found as early as the 23rd day, but the tumors formed by the SiHa-Slug cell group could not be found until the 40th day (Figure 3A), suggesting that the Slug protein may delay the initiation of cervical carcinoma. The volume of the tumors formed by the SiHa-Slug cells was much smaller than that of the tumors formed by the SiHa-GFP control cells (Figure 3A, *p* < 0.05). In addition, the average weight of the tumors formed by the SiHa-Slug cells was much smaller than that of the tumors formed by the SiHa-GFP control cells (Figure 3B, *p* < 0.05), indicating that the over-expression of the Slug protein could suppress tumor initiation and the development of the SiHa cervical cancer cell line *in vivo*. Furthermore, the HeLa-shSlug cells could xenograft the tumors earlier (11th day in the HeLa-shSlug cell group and 13th day in the HeLa-shcontrol group) and could develop much larger (Figure 3C, *p* < 0.05) and heavier tumors (Figure 3D, *p* < 0.01) than the HeLa-shcontrol cells, indicating that the knockdown of Slug in HeLa cells could enhance tumor formation *in vivo*. All of these results demonstrated that the Slug protein could inhibit tumor formation by cervical cancer cells *in vivo*.

To determine whether the *in vivo* tumor suppression function of Slug could be attributed to its cell proliferation inhibition ability, immunohistochemistry was used to determine the expression of Slug and the cell proliferation marker Ki67 [39] in the xenografted cervical cancer tissues. As shown in Figure 3E and 3F, the tumor tissues derived from SiHa-Slug cells expressed much more Slug and less Ki67 than the tumor tissues derived from SiHa-GFP control cells. In addition, the tumor tissues derived from HeLa-shSlug cells expressed less Slug and much more Ki67 than the tumor tissues derived from HeLa-shcontrol cells (Figure 3G and 3H). These results indicated that the expression of Slug adversely affects the cell proliferative ability of cervical cancer cells *in vivo*. These results are consistent with the results obtained from the *in vitro* experiment in this study, suggesting that Slug affects tumor formation by cervical cancer cells *in vivo* in a manner that is dependent on its effects on cell proliferation.

### Slug arrests cervical cancer cells at the transition from the G0/G1 phase to the S phase of the cell cycle

**Figure 4:**
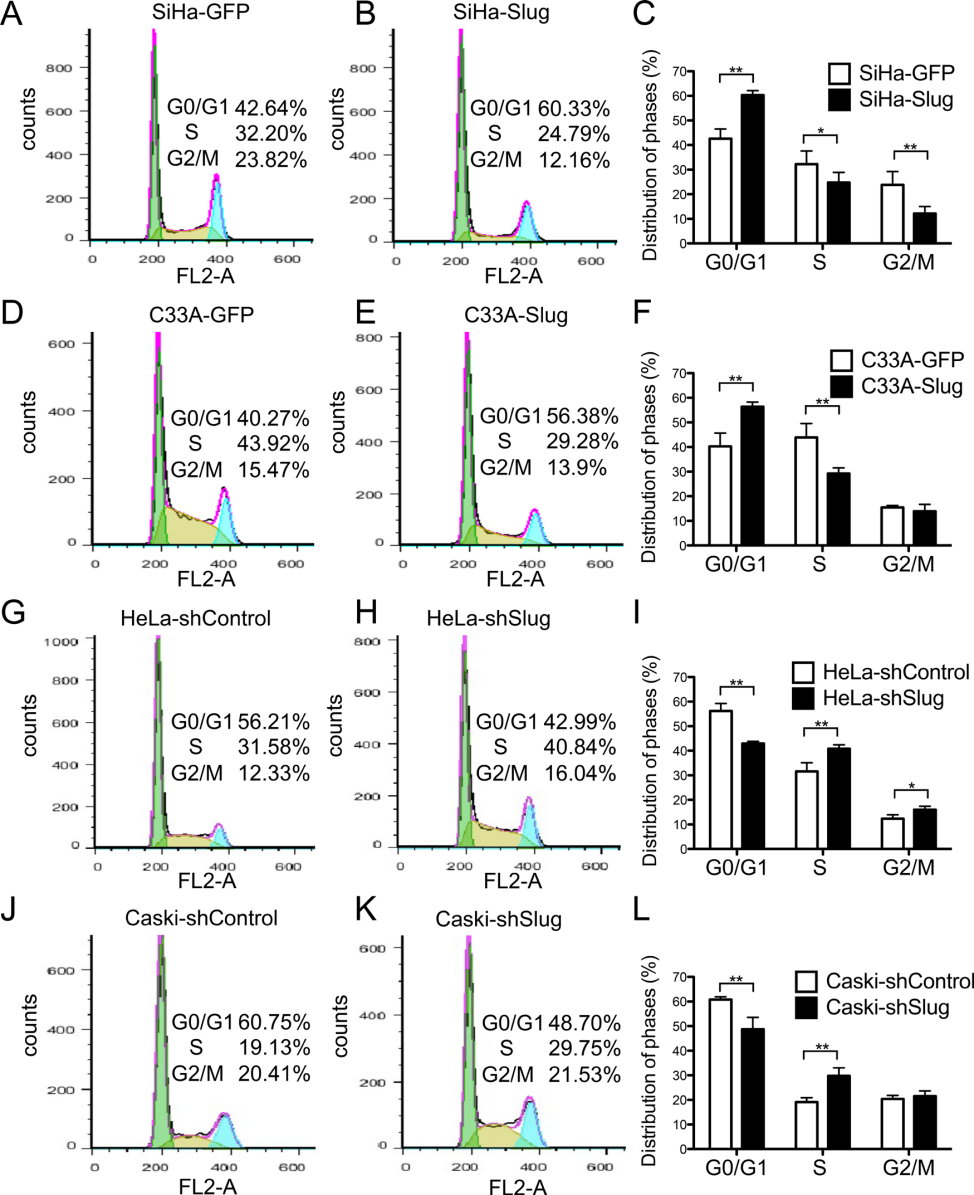
Expression of slug in cervical cancer cells hindered cell cycle transition from G1 to S phase The cell cycle was analyzed using flow cytometry, and a quantitative analysis of the cell cycle is shown. The cell cycles of SiHa-GFP (**A**) and SiHa-Slug cells (**B**) and the quantitative analysis are shown (**C**). The cell cycles of C33A-GFP (**D**) and C33A-Slug cells (**E**) and the quantitative analysis are shown (**F**). The cell cycles of HeLa-shControl (**G**) and HeLa-shSlug cells (**H**) and the quantitative analysis are shown (**I**). The cell cycles of Caski-shControl (**J**) and Caski-shSlug cells (**K**) and the quantitative analysis are shown (**L**). The data were shown as the mean ± SD of three independent experiments. *p < 0.05, **p < 0.01 *vs*. control using One-Way ANOVA.

Generally, the changes that occur during cell proliferation involve the modulation of the cell cycle. To investigate how Slug affects the cell cycle of cervical cancer cells, fluorescence-activated cells sorting (FACS) was used to analyze the differences in the cell cycle between the Slug-modified cells and their control cervical cancer cells. As shown in (Figure 4A, 4B and 4C), the percentage of cells in G0/G1 phase was much higher in the SiHa-Slug cells (60.33%) than in the SiHa-GFP control cells (42.64%), and the percentage of cells in S phase was lower in the SiHa-Slug cells (24.79%) than in the SiHa-GFP control cells (32.20%). The ratio of cells in G1/S phase was much higher in the SiHa-Slug cells (60.33%/24.79%, 2.43) than in the SiHa-GFP cells (42.64%/32.20%, 1.32). A similar result was observed in the C33A cells, and the ratio of cells in the G1/S phase (56.38%/29.28%, 1.93) was much higher in the C33A-Slug cells than in the C33A-GFP cells (40.27%/43.92%, 0.92). These results suggested that the over-expression of Slug induced cell cycle arrest during the G1/S phase transition in the SiHa-Slug and C33A-Slug cells. Conversely, the knockdown of Slug led to a decrease in the G1/S ratio in both HeLa-shSlug (42.99%/40.80%, 1.05; Figure 4G, 4H and 4I) and CasKi-shSlug cells (48.70%/29.75%, 1.64; Figure 4J, 4K and 4L) in comparison to the ratios observed in the HeLa-control cells (56.21%/31.58%, 1.78; Figure 4G, 4H and 4I) and the CasKi-control cells (60.71%/19.13%, 3.17; Figure 4J, 4K and 4L). This result suggested that the knockdown of Slug promoted G1/S phase transition in both HeLa-shSlug and CasKi-shSlug cells. All of these results indicated that Slug inhibited cervical cancer cells at the transition from the G0/G1 phase to the S phase of the cell cycle.

### Slug suppressed the proliferation of cervical cancer cells by up-regulating the p21/p27 proteins and down-regulating Wnt/β-catenin pathway activity via the trans-suppression of Akt1/p-Akt1

**Figure 5:**
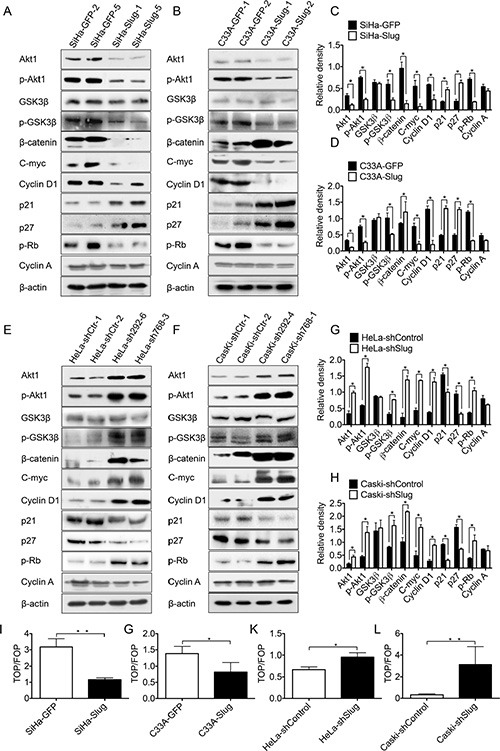
Slug down-regulated Akt1/p-Akt1 expression and suppressed Wnt/β-catenin pathway activity (**A**) The expression of Akt1, p-Akt1, p21, p27, p-RB, p-GSK3β, GSK3β, β-catenin, c-myc, cyclinD1 and cyclinA in SiHa-GFP and SiHa-Slug cells was detected by western blotting, and the quantitative analysis is shown (**C**). (**B**) The expression of Akt1, p-Akt1, p-RB, p21, p27, p-GSK3β, GSK3β, β-catenin, c-myc, cyclinD1 and cyclinA in C33A-GFP and C33A-Slug cells was detected by western blotting, and the quantitative analysis is shown (**D**). (**E**) The expression of Akt1, p-Akt1, p21, p27, p-RB, p-GSK3β, GSK3β, β-catenin, c-myc, cyclinD1 and cyclinA in HeLa-shControl and HeLa-shSlug cells was detected by western blotting, and the quantitative analysis is shown (**G**). (**F**) The expression of Akt1, p-Akt1, p21, p27, p-RB, p-GSK3β, GSK3β, β-catenin, c-myc, cyclinD1 and cyclinA in Caski-shControl and Caski-shSlug cells was detected by western blotting, and the quantitative analysis is shown (**H**). SiHa (**I**), C33A (**G**), HeLa (**K**) and Caski (**L**) cells were transfected with the TOP-Flash reporter plasmid, and the reporter activities were determined 48 h after transfection using a luciferase assay. The data were shown as the mean ± SD of three independent experiments. *p < 0.05, **p < 0.01 *vs*. control using One-Way ANOVA.

It has been demonstrated that Slug can be trans-activated through the PI3K/Akt1 signaling pathway in various cancers [40–43]. However, to our knowledge, no available reports determined whether Slug could regulate the expression of Akt1. Akt1 is a very important protein that strongly affects the cell cycle [44–46]. Therefore, the expression of Akt1/p-Akt1 was detected in the Slug-modified cervical cancer cells and their controls by western blotting. As shown in Figure 5, the Akt1 and p-Akt1 proteins were expressed at much lower levels in both SiHa-Slug (Figure 5A and 5C, *P* < 0.05) and C33A-Slug cells (Figure 5B and 5D, *P* < 0.05) than in SiHa-GFP and C33A-GFP cells, respectively. Furthermore, the Akt1 and p-Akt1 proteins were expressed at much higher levels in the HeLa-shSlug (Figure 5E and 5G, *P* < 0.05) and CasKi-shSlug cells (Figure 5F and 5H, *P* < 0.05) than in HeLa-shControl and CasKi-shControl cells, respectively. All of these data indicated that Slug could trans-suppress the expression of Akt1/p-Akt1 in cervical cancer cell lines.

Akt1 has been demonstrated to be a negative regulator of the cell cycle suppressors p21 and p27 [47–49]. Thus, western blotting analysis was also used to detect the expression of p21 and p27 in the Slug-modified cervical cancer cell lines and their control cells. As shown in Figure 5, the protein levels of p21 and p27 were much higher in the SiHa-Slug (Figure 5A and 5C, *P* < 0.05) and C33A-Slug cells (Figure 5B and 5D, *P* < 0.05) than in the SiHa-GFP cells and C33A-GFP cells, respectively. Furthermore, the expression levels of p21 and p27 were much lower in the HeLa-shSlug (Figure 5E, and 5G, *P* < 0.05) and CasKi-shSlug cells (Figure 5F and 5H, *P* < 0.01) than in the HeLa-shControl and CasKi-shControl cells, respectively. All of these results suggested that Slug could inhibit the proliferation of cervical cancer cells by up-regulating the p21/p27 proteins by trans-suppressing the expression of the Akt1/p-Akt1 proteins.

It has been reported that GSK3β can be phosphorylated through the PI3K/Akt pathway, and GSK3β is a commonly investigated molecular intersection between the Wnt/β-catenin and PI3K/Akt signaling pathways [50, 51]. To determine whether the Wnt/β-catenin signaling pathway could be affected by the PI3K/Akt signaling pathway in cervical cancer cell lines, the TOP-Flash reporter assay was used to identify the activity of the Wnt/β-catenin signaling pathway in Slug-modified cervical cancer cells. As shown in Figure 5, the values obtained in the TOP/FOP assay were much lower in the SiHa-Slug Figure 5I) and C33A-Slug cells (Figure 5G) than in the SiHa-GFP and C33A-GFP cells, respectively. Similarly, the values obtained in the TOP/FOP assay were much higher in the HeLa-shSlug (Figure 5K) and CasKi-shSlug cells (Figure 5L) than in the HeLa-shControl and CasKi-shControl cells, respectively. These data suggested that the activity of the Wnt/β-catenin signaling pathway was attenuated in all Slug-expressing cervical cancer cells.

Moreover, the expression of signaling molecules of the Wnt/β-catenin signaling pathway [52–54], including GSK3β, p-GSK3β, β-catenin, cyclin D1 and c-myc, was detected in the Slug-modified cervical cancer cells and their controls. The protein levels of p-GSK3β, β-catenin, cyclin D1 and c-myc were much higher in the HeLa-shSlug (Figure 5E and 5G, *P* < 0.05) and CasKi-shSlug cells (Figure 5F and 5H, *P* < 0.01) than in the HeLa-shControl and CasKi-shControl cells, respectively. Furthermore, the protein levels of p-GSK3β, β-catenin, cyclin D1 and c-myc were much lower in the SiHa-Slug cell than in the SiHa-GFP cells (Figure 5A and 5C, *P* < 0.05). However, there was no significant difference in GSK3β protein levels between the Slug-modified cells and their control cervical cancer cells. All of these results suggested that Slug negatively regulates the activity of the Wnt/β-catenin signaling pathway in these cervical cancer cell lines. Similarly, the protein levels of p-GSK3β, cyclin D1 and c-myc were also found to be much lower in the C33A-Slug cells than in the C33A-GFP cells (Figure 5B and 5D, *P* < 0.05). However, the protein level of β-catenin was much higher in the C33A-Slug cells than in the C33A-GFP cells (Figure 5B and 5D, *P* < 0.05). Further immunohistochemistry analysis showed that β-catenin accumulated in the cytomembrane but not in the cell nucleus in C33A-Slug cells (Figure S1A). This finding may explain why the C33A-Slug cells exhibited higher protein levels of β-catenin but lower activities of the Wnt/β-catenin signaling pathway in the TOP-Flash reporter assay. All of these results further confirmed that the activity of the Wnt/β-catenin signaling pathway was suppressed in all Slug-expressing cervical cancer cell lines. Cyclin D1 is a key factor that contributes to the cell cycle transition from G1 to S phase [55] and is also an effector of the Wnt/β-catenin signaling pathway [56]. Therefore, in the Slug-modified cervical cancer cells, cell proliferation and tumor formation should be affected by the cyclin D1 protein through the Wnt/β-catenin signaling pathway via the trans-suppression of the expression of the Akt1/p-Akt1 proteins.

The retino-blastoma protein (Rb) is a key factor that blocks cell cycle transition from G1 to S phase. Phosphorylation-Rb (p-Rb) is an inactivation form of Rb, p-Rb protein could be stimulated by cyclinD-CDK4/6 complexes, and results in release of the transcription factor E2F1 in order to activate S-phase entry [57]. Thus, western blotting analysis was used to detect the expression of p-Rb in the Slug-modified cervical cancer cell lines and their control cells. As shown in Figure 5, the protein levels of p-Rb were much lower in the SiHa-Slug (Figure 5A and 5C, *P* < 0.05) and C33A-Slug cells (Figure 5B and 5D, *P* < 0.05) than in the SiHa-GFP cells and C33A-GFP cells, respectively. Furthermore, the expression levels of p-Rb were much higher in the HeLa-shSlug (Figure 5E, and 5G, *P* < 0.05) and CasKi-shSlug cells (Figure 5F and 5H, *P* < 0.01) than in the HeLa-shControl and CasKi-shControl cells, respectively. All of these results indicated that Slug could inhibit cervical cancer cells at the transition from the G0/G1 phase to the S phase of the cell cycle.

**Figure 6:**
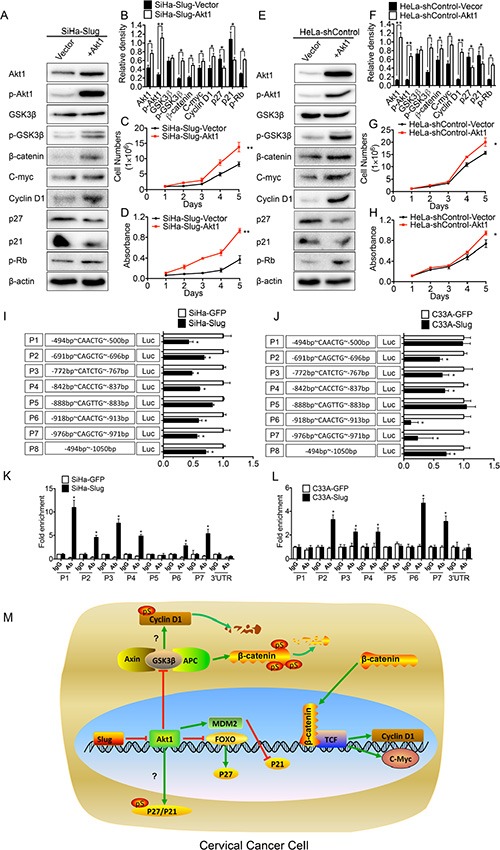
Slug trans-suppressed Akt1/p-Akt1 (**A**) The expression of Akt1, p-Akt1, p21, p27, p-RB, p-GSK3β, GSK3β, β-catenin, c-myc and cyclinD1 in Akt1 transiently transfected SiHa-Slug cells was detected by western blotting, and the quantitative analysis was shown (**B**). The proliferation and viability of Akt1 transiently transfected SiHa-Slug cells were detected by growth curves (**C**) and MTT assay (**D**). (**E**) The expression of Akt1, p-Akt1, p21, p27, p-RB, p-GSK3β, GSK3β, β-catenin, c-myc and cyclinD1 in Akt1 transiently transfected HeLa-shControl cells was detected by western blotting, and the quantitative analysis was shown (**F**). The proliferation and viability of Akt1 transiently transfected HeLa-shControl cells were detected by growth curves (**G**) and MTT assay (**H**). (**I**) The activity of the Akt1 promoter was measured using the dual luciferase assay and presented as the fold change in the rate of SiHa-Slug cells versus SiHa-GFP cells. (**J**) The activity of the Akt1 promoter was measured using the dual luciferase assay and presented as the fold change in the rate of C33A-Slug cells versus C33A-GFP cells. (**K**) A quantitative CHIP assay of the Akt1 promoter region in SiHa-Slug and SiHa-GFP cells is shown. (**L**) A quantitative CHIP assay of the Akt1 promoter region in C33A-Slug and C33A-GFP cells is shown. (**M**) Proposed model of the Slug-mediated disruption of Akt1/p-Akt1 and Wnt/β-catenin signaling in cervical cancer cells. When exogenous Slug was expressed in cervical cancer cells, Slug could recognize and bind to the E-boxes in the Akt1 promoter region as a transcription repressor and reduce Akt1/p-Akt1, leading to the up-regulation of p21 and p27. In addition, the reduction of Akt1/p-Akt1 also attenuated the Wnt/β-catenin signaling pathway and suppressed cell proliferation and tumor formation in cervical cancer cells through GSK3β. The data were shown as the mean ± SD of three independent experiments. *p < 0.05, **p < 0.01 *vs*. control using One-Way ANOVA.

To further confirm that Slug inhibited cell proliferation and tumor formation of cervical cancer cells by trans-suppressing Akt1/p-Akt1 through both the p21/p27 proteins and the Wnt/β-catenin signaling pathway, first of all, the recombinant Akt1 plasmid was transiently transfected in the SiHa-Slug and HeLa-shControl cells, in which high level of Slug expression trans-suppressed the expression of Akt1/p-Akt1. As shown in Figure 6, the SiHa-Slug and HeLa-shControl cells transfected by pIRES2-AcGFP-Akt1 grew much faster than the cells transfected with the control vector, respectively (Figure 6C, 6D, 6G and 6H, *P* < 0.05). Simultaneously, the SiHa-Slug and HeLa-shControl cells transfected by pIRES2-AcGFP-Akt1 also express more Akt1/p-Akt1, less p21/p27, more p-Rb and more molecules (p-GSK3β, β-catenin, c-myc and cyclin D1) of the Wnt/β-catenin signaling pathway than the cells transfected with the control vector, respectively (Figure 6A, 6B, 6E, and 6F, *P* < 0.05). Secondly, MK-2206 (MK-2206), which is an inhibitor of Akt1 [58], was used in all of the Slug-modified the cervical cancer cells investigated in this study. All of the cervical cancer cells subjected to the MK treatment grew much more slowly and expressed less Akt1/p-Akt1, more p21/p27, less p-Rb and fewer molecules (p-GSK3β, β-catenin, c-myc and cyclin D1) of the Wnt/β-catenin signaling pathway than the cells that were not subjected to MK treatment (Figure S2). All of these results further confirmed that Slug inhibited cell proliferation and tumor formation of human cervical cancer cells via trans-suppressing Akt1/p-Akt1 through both the p21/p27 proteins and the Wnt/β-catenin signaling pathway.

### Slug trans-suppressed Akt1/p-Akt1 expression by binding to the E-box motifs in the Akt1 promoter region

Dr. ER Fearon demonstrated that the E-box CACCTG motif was a binding site in the E-cadherin promoter region that allowed Slug to recognize and bind [25] to trans-suppress the expression of E-cadherin. A sequence analysis through the UCSC Genome online database revealed that seven E-boxes (CANNTG) are clustered in the Akt1 promoter region (from −494 bp to −1050 bp): one CACCTG (P4), two CAACTG (P1, P6), two CAGCTG (P2, P7), one CATCTG (P3), and one CAGTTG (P5). A luciferase reporter assay was used to determine whether Slug could trans-suppress the expression of Akt1 through these E-boxes (CANNTG). As shown in Figure 6I and 6J, E-boxes P2, P3, P4, P6 and P7 in the Akt1 promotor region in both SiHa-Slug and C33A-Slug cells had significantly stronger trans-suppression activities than their respective control cells (Figure 6I and 6J; *p* < 0.05), suggesting that Slug could trans-suppress Akt1/p-Akt1 expression through the E-boxes in the Akt1 promoter region in cervical cancer cells.

Furthermore, a chromatin immunoprecipitation assay (ChIP) was used to determine whether Slug could recognize and bind directly to the E-boxes in the Akt1 promoter region. And the E-box CACCTG motif in the E-cadherin promoter region was used as a positive control (Figure S1F and S1G; *p* < 0.05). As shown in Figure 6K and 6L, Slug could specifically recognize and bind to the P2, P3, P4, P6 and P7 E-boxes in the Akt1 promoter region in both SiHa-Slug and C33A-Slug cells (Figure 6K and 6L; *p* < 0.05). All of these data indicated that Slug could recognize and bind to the E-boxes in the Akt1 promoter region and trans-suppress the expression of Akt1/p-Akt1.

## DISCUSSION

Slug is known to participate in the epithelial to mesenchymal transition (EMT) [22–24], and it is also been found to have different effects on cell proliferation and tumor formation in different carcinomas. But to our knowledge, no available reports identified the function of Slug in cervical carcinoma. As shown in Figure 1, both the immunoreactivity scores and western blotting analysis revealed that the expression of Slug was lower in cervical carcinoma tissues than in normal cervix tissues, suggesting that Slug is involved in the suppression of the development of cervical carcinoma.

When exogenous Slug protein was expressed in the SiHa and C33A cell lines (Figure 2A and 2B), *in vitro* cell proliferation and *in vivo* tumor formation were inhibited (Figure 2 and Figure 3). Moreover, *in vitro* cell proliferation and *in vivo* tumor formation were promoted (Figure 2 and Figure 3) when endogenous Slug protein expression was down-regulated in the HeLa and CasKi cell lines (Figure 2A and 2B). Therefore, Slug works as a cell proliferation inhibitor and tumor suppressor in cervical carcinoma, regardless of the levels of endogenous Slug protein in the cervical carcinoma cells. Furthermore, cell cycle analysis also showed that Slug arrested cell proliferation at the cell G1/G0 transition in all Slug-modified cervical carcinoma cells (Figure 4). Therefore, Slug appeared to inhibit cell proliferation and tumor formation by regulating G0/G1 transition-related cell cycle proteins.

There are several main G0/G1 transition-related cell cycle proteins: the cyclin D1, Rb, p21 and p27 proteins [59–61]. Dr. WS Wu demonstrated that Slug could inhibit cell proliferation in human prostate cancer cells via the down-regulation of cyclin D1 [38] but did not show how Slug down-regulated cyclin D1 expression in human prostate cancer cells. The cyclin D1 protein is a down-stream protein of the Wnt/β-catenin signaling pathway, and its expression is correlated positively with the activity of the Wnt/β-catenin signaling pathway [56]. Moreover, the cyclin D1 protein may also be degraded by GSK3β [62]. Akt1 was reported to be able to trans-activate Slug expression to promote EMT progression in breast cancer and melanoma cells [41, 43], but no data revealed whether Slug could regulate Akt1. In the present study, we demonstrated that Slug could trans-suppress Akt1/p-Akt1 expression (Figure 5, *p* < 0.05) in cervical carcinoma cells and then decrease the phosphorylation of GSK3β (Figure 5, *p* < 0.05) by Akt1. Moreover, active GSK3β increased the degradation of β-catenin and down-regulated the activity of the Wnt/β-catenin signaling pathway (Figure 5, *p* < 0.05) and the expression of related molecules, such as β-catenin, c-myc and cyclin D1 (Figure 5, *p* < 0.05). Moreover, mRNA levels of cyclin D1 were found to be much lower in the SiHa-Slug, C33A-Slug, HeLa-shControl and CasKi-shControl cells than in the SiHa-GFP, C33A-GFP, HeLa-shSlug and CasKi-shSlug cells, respectively (Figure S1B, S1C, S1D and S1E; *P* < 0.05), indicating that Slug could suppress cell proliferation and tumor formation through the Akt1/p-Akt1/GSK3β/Wnt/β-catenin signaling pathway/cyclin D1 in cervical carcinoma. Of course, further experiments will be required to confirm whether the down-regulation of cyclin D1 by Slug in cervical carcinoma cells also occurs through direct degradation by GSK3β at the protein level [62].

Additionally, we demonstrated that Slug could trans-suppresses Akt1/p-Akt1 protein expression and simultaneously up-regulates p21/p27 protein expression in all cervical cancer cells (Figure 5, *p* < 0.05). Akt1 has been found to be able to down-regulate the p21 protein at the transcriptional level through MDM2/p53 [63] or to down-regulate the p27 protein at the transcriptional level through FOXO [59]. Moreover, quantitative real-time–PCR results suggested that Slug could suppress cell proliferation and tumor formation through the up-regulation of p21/p27 at the transcriptional level through MDM2/p53 and FOXO (Figure S1B, S1C, S1D and S1E; *P* < 0.05). However, further experiments will be required to determine whether the up-regulation of p21/p27 by Slug is also caused by the decreased phosphorylation of p21/p27 through the trans-suppression of Akt1/p-Akt1 in cervical carcinoma cells.

Furthermore, Luciferase assays and CHIP assays confirmed that Slug could recognize (Figure 6I and 6J) and bind (Figure 6K and 6L) to the E-boxes in the Akt1 promoter region and act as a transcription repressor to reduce Akt1/p-Akt1 expression in both SiHa-Slug and C33A-Slug cells (Figure 6I, 6J, 6K and 6L). This is the first study to demonstrate that Slug could trans-suppress Akt1/p-Akt1 expression through the E-boxes in the Akt1 promoter region.

In conclusion, this is the first study to demonstrate that Slug may trans-suppress Akt1/p-Akt1 expression by binding to the E-boxes in the Akt1 promoter region and inhibit the proliferation and tumor formation of human cervical cancer cells by up-regulating p21/p27 and/or down-regulating the activity of the Wnt/β-catenin signaling pathway (Figure 6M). Slug was found to act as a tumor suppressor in cervical carcinogenesis.

## MATERIALS AND METHODS

### Cell lines and human tissue specimens

Human cervical carcinoma cell lines SiHa, C33A, HeLa, CasKi and HT-3 were purchased from the American Type Culture Collection (ATCC, Rockville, MD, USA). SiHa, C33A and HeLa were cultured in high-glucose Dulbecco Modified Eagle Medium (DMEM, Sigma-Aldrich, St Louis, MO, USA), CasKi was cultured in RPMI1640 (Sigma-Aldrich, St Louis, MO, USA), HT-3 was cultured in McCoy's 5A Medium (Sigma-Aldrich, St Louis, MO, USA), and the cell culture mediums contained 10% fetal bovine serum (FBS, Invitrogen, Carlsbad, CA, USA). Cell lines were all incubated at 37°C in a humidified 5% CO_2_ atmosphere.

A total of 37 normal cervixes (NC), 28 cervical carcinomas *in situ* (CIS), and 52 squamous cervical cancer (SCC) tissues were obtained from the First Affiliated Hospital of Xi'an Jiaotong University between January 2008 and December 2014. 8 normal cervixes and 8 squamous cervical cancers (SCC) fresh tissues were collected from the First Affiliated Hospital of Xi'an Jiaotong University for Western blot analysis.

### Immunohistochemistry and immunocytochemistry

The immunohistochemical staining procedure was performed as previously described. Briefly, four-millimeter sections of formalin-fixed and paraffin-embedded tissue specimens were deparaffinized in xylene and rehydrated through descending concentrations of ethanol. After antigen retrieval was performed by heating in 10 mM citrate buffer (pH 6.0) for 2 min, the sections were treated with 3% hydrogen peroxide to block endogenous peroxidase. Subsequently, the sections were incubated with a primary antibody overnight at 4°C. A horseradish peroxidase-conjugated secondary antibody was added for 30 min at room temperature, followed by 3, 3′-diaminobenzidine development. The sections were counterstained with hematoxylin. As a negative control, the primary antibody was replaced with PBS.

A standard immunostaining procedure was performed using a rabbit polyclonal antibody against human Slug (1:50 dilution; #9585, Cell Signaling Technology). A positive reaction was defined as the observation of a reddish-brown precipitate in the nucleus. Slug staining was classified into 2 categories (negative and positive expression) based on the percentage of positive cells and the staining intensity [64]. The percentage of positive cells was divided into 5 scores: < 5% (0), 5% to 25% (1), 25% to 50% (2), 50% to 75% (3), and > 75% (4). The intensity of staining was divided into 4 scores: no staining (0), light brown (1), brown (2), and dark brown (3). The positivity of Slug staining was determined using the following formula: immunohistochemistry (IHC) score = percentage score × intensity score. An overall score of ≤ 1 was defined as negative; a score of ≥ 2 was defined as positive.

For the expression of Slug in cells, similar immunocytochemistry was performed after the cells were seeded onto cover slips for 48 hours, fixed with 4% paraformaldehyde for 20 minutes, and permeabilized with 0.2% Triton X-100 for 20 minutes at room temperature.

### Western blotting

Western blotting analysis was carried out as previously described [65], the rabbit polyclonal antibody against human Slug (1:1000 dilution; #9585, Cell Signaling Technology), β-actin (1:1000 dilution; Santa Cruz, CA, USA), Akt1 (1:1000 dilution; Santa Cruz, CA, USA), p-Akt1 (1:1000 dilution; Cell Signaling Technology), p21 (1:500 dilution; Santa Cruz, CA, USA), p27 (1:500 dilution; Santa Cruz, CA, USA), cyclin A (1:1000 dilution; Santa Cruz, CA, USA), p-Rb (1:500 dilution; Santa Cruz, CA, USA), p-GSKβ (1:1000 dilution; Santa Cruz, CA, USA), β-catenin (1:1000 dilution; Santa Cruz, CA, USA), c-myc (1:1000 dilution, Santa Cruz, CA, USA), and cyclin D1 (1:500 dilution, Santa Cruz, CA, USA) were incubated with the membranes at 4°C overnight, followed by secondary incubation using a horseradish peroxidase-conjugated anti-rabbit or anti-mouse IgG (Thermo Fisher Scientific, New York, NY, USA). The proteins were visualized with an enhanced chemiluminescence reagent (Millipore, Billerica, MA, USA) through the protein imprinting imaging system (Tanon 5200, China). The Slug western blot results were normalized to those of β-actin blotting for quantification.

### Cell growth and cell viability assays

Cells (5 × 10^4^) were seeded in 2-mL media in 6-well plates in triplicate. Then the cells were counted every 2 days for 1 week using hemocytometer. Cell growth curves were generated to assess the cell proliferation. Cell viability was assessed using 3-(4, 5-dimethylthiazole-yl)-2, 5-diphenyl tetrazolium bromide (Sigma-Aldrich, St Louis, MO, USA) dye according to standard protocol. The number of viable cells was detected by measuring the absorbance at 490 nm.

### Tumor xenograft assay

We obtained 6- to 7-week-old female BALB/c-nude mice from Slac Laboratory Animal Co., Ltd. (Shanghai, China). The mice were housed in an specific pathogen free (SFP) room with a constant temperature (22°C–25°C) and humidity (40–50%).

Tumor cells in the exponential growth phase were harvested for inoculation, and the tumor cells (1 × 10^5^) were then injected into the subcutis on the dorsum of each female BALB/c-nude mouse. The tumor volume (V) was determined using the following formula: length (a) and width (b) as *V* = ab^2^/2. At the end of the experiment, the tumors were weighed and stored for immunostaining. The experimental protocols were evaluated and approved by the Animal Care and Use Committee of the Medical School of Xi'an Jiaotong University.

### Flow cytometry analysis

Before FACS analysis, 1 × 10^6^ cells were harvested and washed twice with cold PBS and then fixed in 70% ice-cold ethanol overnight at 4°C for thirty minutes. After washing twice with PBS, the cells were treated with RNaseA and stained with propidium iodide (Sigma-Aldrich, St. Louis, MO, USA). Then, the cell was analyzed using a FACS Calibur flow cytometer (BD Biosciences, San Jose, CA, USA) with the CellQuest software.

### PCR analysis

Total RNA from cultured cervical cancer cells was extracted using the TRIzol Reagent (Invitrogen, Carlsbad, CA, USA). Total cDNA was used as a template for PCR amplification, with β-actin as an internal control. Real-time quantitative PCR was performed in triplicate for each primer set and each cell sample, using an IQ5 multicolor real-time PCR Detection System (Bio-Rad, Hercules, CA). The protocol for real-time PCR was 1 cycle of 95 for 30 seconds, 40 cycles of 95°C for 5 seconds and 60 for 30 seconds, and then a dissociation stage. The cycle threshold value was determined as the point at which the fluorescence exceeded a preset limit determined by the instrument's software. The results were analyzed *via* the ΔΔCt method using GAPDH as the housekeeping genes, Primers used were as follows: GAPDH (GCACCGTCAAGGCTGAGAAC and TGGT GAAGACGCCAGTGGA); p27 (CTGCCCTCCCCAGT CTCTCT and CAAGCACCTCGGATTTT); p21 (GCAGA CCAGCATGACAGATTTC and CGGATTAGGGCTTC CTCTTG); and cyclin D1 (AAACAGATCATCCG CAAACAC and GTTGGGGCTCCTCAGGTTC).

### Vector construction and transfection

Full-length Slug cDNA was amplified using the following primers: forward, 5′-GTTGAATTCGTTATG CCGCGCTCCTTCCTG-3′ and reverse, 5′-CGCGGATCC TCAGTGTGCTACACAGCAG-3′. The Slug DNA fragment was subsequently cloned into the EcoRI and BamHI sites of the internal ribosome entry site vector pIRES2-AcGFP (Clontech, Mountain View, CA) to generate the pIRES2-AcGFP-Slug recombinant plasmid. The small interfering RNA expression vector that expresses a Slug-specific short hairpin RNA (shRNA) was purchased from GenePharma (Shanghai, China). The Slug overexpression and shRNA vectors were transfected into SiHa, C33A, HeLa and CasKi cells using the Lipofectamine 2000 reagent (Invitrogen, Carlsbad, CA, USA) according to the manufacturer's protocol. The transfected cells were treated with G418 (Calbiochem, La Jolla, CA, USA) for 3 weeks to collect, expand, and identify the drug-resistant colonies. The recombinant Akt1 plasmid was adapted from a previous study [66].

### Luciferase reporter assay

For promoter analyses, a fragment (from position −494 bp to −1050 bp relative to Akt1) containing the E-box site (CANNTG) was cloned into the pGL3-Basic Vector (Promega, Madison, WI, USA) to generate Akt1 promoter reporter constructs. Plasmids containing firefly luciferase reporters were co-transfected into tumor cells in triplicate using Lipofectamine 2000 (Invitrogen, Carlsbad, CA, USA), with the thymidine kinase promoter Renilla luciferase reporter plasmid (pRL-TK) as an internal control. The activity of both the firefly and Renilla luciferase reporters was determined 48 hours after transfection using the Dual Luciferase Assay kit (Promega, Madison, WI). The specific promoter activity was presented as the relative ratio of firefly luciferase activity to Renilla luciferase activity. The specific promoter activity was presented as the change in the experimental group versus the control group. The primers and oligonucleotides are listed in Table S2. Restriction enzymes were obtained from TaKaRa. All constructs were verified by sequencing. The specific activity is shown as the fold change of the experimental group versus the control group.

### Quantitative chromatin immunoprecipitation

SiHa and C33A cells were subjected to ChIP using the EZ-ChIP Assay kit (Millipore). Briefly, the cells were treated with 37% formaldehyde to crosslink proteins, and the reaction was terminated with 0.125 M glycine. After sonication, chromatin–protein complexes were immunoprecipitated with 5 μg of anti-Slug antibodies (#9585, Cell Signaling Technology) or 1 μg of mouse IgG. Real-time PCR was performed to amplify the regions of interest or internal negative control regions. Each sample was assayed in triplicate, and the fold enrichment ratio was calculated as the value of the ChIP sample versus the corresponding input sample. Samples that yielded a two-fold enrichment or better were considered positive targets. The primers used for these studies are listed in Table S3.

### Statistical analysis

Statistical analysis was performed with SPSS 16.0 software (SPSS Inc., Chicago, IL, USA). The significance of the differences between the covariates was determined by the 2-tailed chi-square test. The Student *t* test was used to determine the statistical significance for 2-group analyses. In all tests, statistically significant was defined as *p* < 0.05.

## SUPPLEMENTARY FIGURES AND TABLES


