# Supplementary material for: Slug inhibits the proliferation and tumor formation of human cervical cancer cells by up-regulating the p21/p27 proteins and down-regulating the activity of the Wnt/β-catenin signaling pathway via the trans-suppression Akt1/p-Akt1 expression
Source: Oncotarget. 2016 Mar 28;7(18):26152–67. doi: 10.18632/oncotarget.8434 (PMC5041971; doi:10.18632/oncotarget.8434)
Supplement: Supplementary file 1 [file oncotarget-07-26152-s001.pdf]

# Slug inhibits the proliferation and tumor formation of human cervical cancer cells by up-regulating the p21/p27 proteins and down-regulating the activity of the Wnt/β-catenin signaling pathway via the trans-suppression Akt1/p-Akt1 expression

## Supplementary Materials

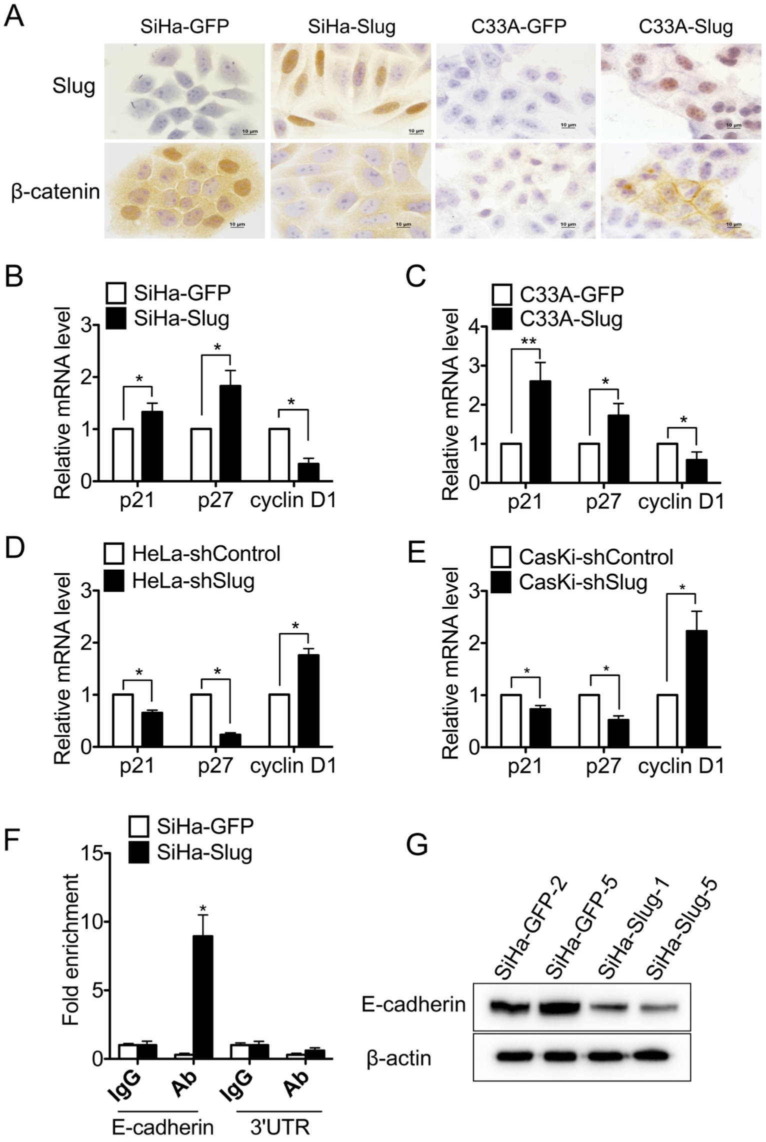


**Supplementary Figure S1: (A)** IHC detection of β-catenin and slug in SiHa-GFP, SiHa-Slug, C33A-GFP and C33A-Slug cells. (**B**) The expression of p21, p27 and cyclinD1 in SiHa-GFP and SiHa-Slug cells was detected by quantitative real-time–PCR. (**C**) The expression of p21, p27 and cyclinD1 in C33A-GFP and C33A-Slug cells was detected by quantitative real-time–PCR. (**D**) The expression of p21, p27 and cyclinD1 in HeLa-shControl and HeLa-shSlug cells was detected by quantitative real-time–PCR. (**E**) The expression of p21, p27 and cyclinD1 in Caski-shControl and Caski- shSlug cells was detected by quantitative real-time–PCR. (**F**) A quantitative CHIP assay of the E-cadherin promoter region in SiHa-Slug and SiHa-GFP cells is shown. (**G**) The expression of E-cadherin in SiHa-Slug and SiHa-GFP cells was detected by western blotting. The data were shown as the mean ± SD of three independent experiments. $^*p < 0.05$, $^{**}p < 0.01$ *vs*. control using One-Way ANOVA.

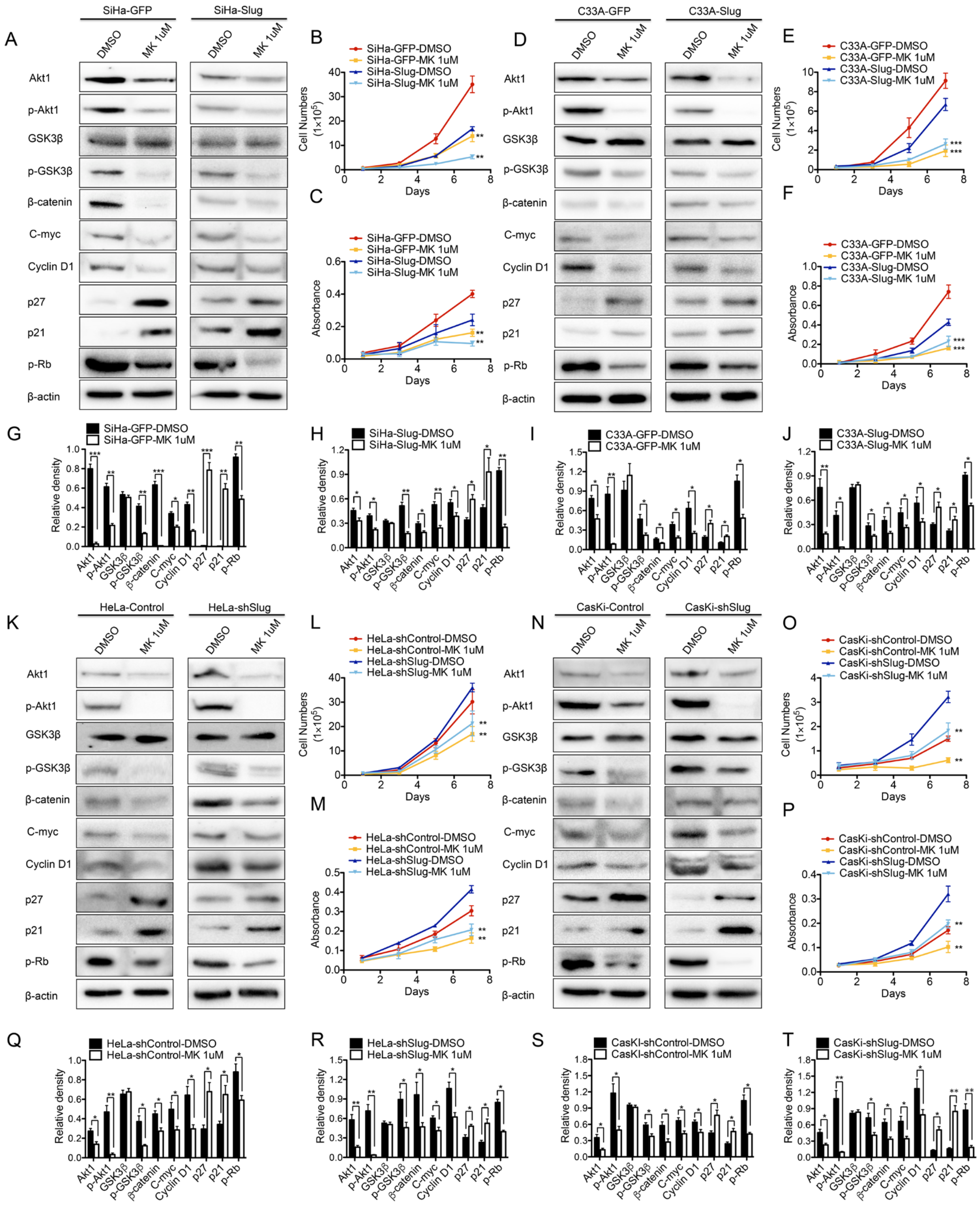


**Supplementary Figure S2: Blockage of the Akt1/p-Akt1 suppresses cell proliferation in the slug-modified cervical cancer cells by MK-2206.** (**A**) The expression of Akt1, p-Akt1, p21, p27, p-Rb, p-GSK3β, GSK3β, β-catenin, c-myc and cyclinD1 in MK-treated SiHa-GFP and SiHa-Slug cells was detected by western blotting, and the quantitative analysis was shown (**G** and **H**). The proliferation and viability of MK-treated SiHa-GFP and SiHa-Slug cells were detected by growth curves (**B**) and MTT assay (**C**). (**D**) The expression of Akt1, p-Akt1, p21, p27, p-Rb, p-GSK3β, GSK3β, β-catenin, c-myc and cyclinD1 in MK-treated C33A-GFP and C33A-Slug cells was detected by western blotting, and the quantitative analysis was shown (**I** and **J**). The proliferation and viability of MK-treated C33A-GFP and C33A-Slug cells were detected by growth curves (**E**) and MTT assay (**F**). (**K**) The expression of Akt1, p-Akt1, p21, p27, p-Rb, p-GSK3β, GSK3β, β-catenin, c-myc and cyclinD1 in MK-treated HeLa-shControl and HeLa-shSlug cells was detected by western blotting, and the quantitative analysis was shown (**Q** and **R**). The proliferation and viability of MK-treated HeLa-shControl and HeLa-shSlug cells were detected by growth curves (**L**) and MTT assay (**M**). (**N**) The expression of Akt1, p-Akt1, p21, p27, p-Rb, p-GSK3β, GSK3β, β-catenin, c-myc and cyclinD1 in MK-treated Caski-shControl and Caski-shSlug cells was detected by western blotting, and the quantitative analysis was shown (**S** and **T**). The proliferation and viability of MK-treated Caski-shControl and Caski- shSlug cells were detected by growth curves (**O**) and MTT assay (**P**). The data were shown as the mean ± SD of three independent experiments. *$p < 0.05$, **$p < 0.01$ *vs*. control using One-Way ANOVA.

## Supplementary Table S1: Slug expression levels in different tissue specimen

| Specimens | Total | Slug Staining | | *P* |
|---|---|---|---|---|
| | | Negative, No. (%) | Positive, No. (%) | |
| Normal | 38 | 4.667 ± 0.5774 (12.69 ± 0.8083) | 33.33 ± 0.5774 (87.72 ± 1.518) | |
| Cancer *in situ* | 24 | 9.667 ± 1.155 (40.28 ± 4.809) | 14.33 ± 1.155 (59.73 ± 4.792) | < 0.05[a] |
| Carcinoma | 52 | 17.67 ± 1.155 (33.96 ± 2.200) | 31.67 ± 1.155 (62.18 ± 2.217) | < 0.05[b] |

Abbreviation: Slug
Pearson 2-tailed chi-square test was used to determine the statistical significance of the level of expression of Slug in different tissue specimens.
[a]Normal cervix versus cervical cancer in situ.
[b]Normal cervix versus carcinoma.

## Supplementary Table S2: The list of primer sequences that used for luciferase assays in this study

| Primer name | Location | F/R | Sequence |
|---|---|---|---|
| **P1** | –585 ~ –355 | F | CGACGCGTCTTTTGTGAGTGTAG |
| | | R | GGAAGATCTTGGCTTAGGTTGACTT |
| **P2** | –767 ~ –511 | F | CGACGCGTTGGACTTCGGACT |
| | | R | GGAAGATCTTGGCAGCTACACTCAC |
| **P3** | –831 ~ –706 | F | CGACGCGTTGTCCAGGAGAAAG |
| | | R | GGAAGATCTTGCTGGGTGGACTTG |
| **P4** | –886 ~ –744 | F | CGACGCGTAGAACTTCTGGCT |
| | | R | GGAAGATCTGGAATGAGTAAGTGG |
| **P5** | –918 ~ –846 | F | CGACGCGTAACTCTGGAATGG |
| | | R | GGAAGATCTACCCCTTCCTAGCC |
| **P6** | –1012 ~ -888 | F | CGACGCGTAATAAAAATGCTCC |
| | | R | GGAAGATCTCCATTCCAGAGGC |
| **P7** | –1116 ~ –923 | F | CGACGCGTATTGGCTGCAGACT |
| | | R | GGAAGATCTCGTGAAAGACAGACTCTTG |
| **P8** | –1345 ~ –221 | F | CGACGCGTAACCCTTGTGTCAGGT |
| | | R | GGAAGATCTTCTCTGGCCTCAGTTTC |

**Supplementary Table S3: The list of primer sequences that used for chromatin immunoprecipitation assay (ChIP) in this study**

| Primer name | Location | F/R | Sequence |
|---|---|---|---|
| **P1** | –534 ~ –462 | F | TCAAAGCCTTCCTGCTCCTT |
| | | R | AAGGAAGTGCGGGAGGAT |
| **P2** | –738 ~ –644 | F | AGGCTGACCAAGTCC |
| | | R | GAGCAGACACCAGACAG |
| **P3** | –824 ~ –717 | F | GTGTCCAGGAGAAAGG |
| | | R | ATGGGTGGACTTGGTC |
| **P4** | –876 ~ –788 | F | AACTTCTGGCTAGGAAGG |
| | | R | GAGTAAGTGGGACACAGAC |
| **P5** | –915 ~ –858 | F | TGTGGGCCTCTGGAATG |
| | | R | CCTTCCTAGCCAGAAGTTC |
| **P6** | –982 ~ –900 | F | CAATACTTAGCAGCCTCAGG |
| | | R | TTCCAGAGGCCCACAGTT |
| **P7** | –1068 ~ –985 | F | CTGCCTCTGTCTGCATCT |
| | | R | TTGGGGGAGCATTTTTAT |
| **3′UTR** | Akt1 (3′UTR) | F | CGTTTTTGTGCTGTGGGC |
| | | R | CATTTCCCTACGTGAATCG |
| **E-cadherin** | –25 ~ 110 | F | CGTCGGAACTGCAAAGC |
| | | R | TATGTGCGGTCGGGTCG |
